# A Proposal for a Work on the Therapeutics of the Throat

**Published:** 1887-06

**Authors:** E. B. Nash

**Affiliations:** Cortland, N. Y.


### A PROPOSAL FOR A WORK ON THE THERAPEUTICS OF THE THROAT.

E. B. Nash, M. D., Cortland, N. Y.

I have received urgent invitations to contribute something to the pages of our homœopathic medical journals. I have sometimes been able to respond, but oftener not. No one knows better than the *reader* of the journals (barring the editors, perhaps) how difficult it must be to suit the individual tastes that go to make up the sum of the homœopathic brotherhood. And now that we have so many degrees of Homœopathy represented in our school, from the coarsest eclecticism to the most exact and conscientious Hahnemannianism, the task becomes even more difficult.

I would not feel competent, or be ambitious to assume the role of dictator to our editors and thus make myself responsible (or at least be called to account) for the foolishness and disloyalty to principle that sometimes creeps into their pages; as Josiah Allen's wife says, "I find it a mighty tuckering job to take care of one poor sinner," let alone being responsible for my neighbors.

Now that I have begged off from the above-mentioned, may I be pardoned if I suggest something that I think may add to the interest and usefulness of our journals, something *practical.* When I say practical, I mean something that may be utilized in everyday work. Do not understand me to say or think that we have not already some such articles. Such articles as Carlton's on Xanthoxylum and Lippe's on Tellurium are instances of the kind, and it is in this line of materia medica and therapeutics, which distinguishes our school permanently from all others, that the *practical* part of the business lies.

Of course, we have continual and increasing need, in these times of mongrelism and false teaching, of those writers who can thunder like Demosthenes, or persuade like Cicero for the great and pure principles which underlie our divine art of healing. But, after all, as the Irishman said, the proof of the pudding is in the ateing of it. So the proof of the *excellence* of our system is in the *application* of it, and we need the most ample and exact means of making that application.

Who that has used Bell on Diarrhœa, Lee on Cough, Eggert or Minton on Uterine Therapeutics, but is ready to "rise up and call their authors blessed"?

But it takes a long time for one man to produce such a work, and few out of or in a busy practice are able or willing to undertake it. Then how can we get such works? Now, *my proposition:*

Let us have a work on Therapeutics of the Throat. "A good many hands make light work." I contribute my part to it in working up Phytol. dec. in a way that looks to me to be a practical one, and invite the kindly criticism or suggestions in the way of additions, comparisons, etc., from my brethren. Now let the editors of THE PHYSICIAN call upon, for instance Lippe for Lachesis, P. P. Wells for Lycop., Carlton for Bell., Berridge for Lac. can., and so on for a long list of remedies having prominent action and curative power on the throat. Who would not be willing to contribute *so* much, if called upon, to such a good work?

To avoid confusion, let the editors of the journal call upon the men and assign to each his remedy.

Let the names of those who consent to work out a remedy be announced in the journal, so that no two will be working out the same one at a time.

Now let it be published in the journal and paged separately, and in one year or less we will be able to do more for throat affections than ever before.

Finally, let some good and competent man (say Wm. J. Guernsey), as the remedies appear in print, arrange them into the form of a repertory, to be bound all together like Bell on Diarrhœa, and the thing is done, and, except the repertory part, a good many hands (and heads) have contributed to make *light work* in the production of a *practical* one.

Here is, for a beginning, my contribution on

### Phytolacca Decandra—Objective.

Fauces and tonsils swollen; dark red, sometimes bluish-red. Tonsils, soft palate, and fauces highly congested or inflamed, very much swollen, sore and sensitive, of a dark-purple color. Ulcerated tonsils; uvula large, transparent. Isthmus congested and of a dark color. Little white or yellow spots appear upon the tonsils, which soon coalesce and form patches of grayish white or yellow membrane. Exudation mostly of a grayish color, or a livid exudation upon fauces and tonsils. Dirty wash-leather, pseudo-membrane. Mucus hawked with difficulty from posterior nares or hangs down in strings.

### Subjective.

Soreness of the throat and a feeling when swallowing saliva as if a lump had formed there. The same sensation is felt on turning the head to the left side.

Sensation of rawness and scraping in the throat and tonsils, as if something had lodged at the root of the tongue. A sensation of the throat being so full that it felt choked; hawking to rid the throat and posterior nares of mucus; pressing pain in the right side of the throat.

The throat feels very dry and sore, especially on swallowing in the morning, with feeling of lump there; like a plug in the throat—worse left side. Sensation as if a hot substance were continually there, or as though a red-hot ball was lodged in the fauces. Granular pharyngitis (chronic), with this same hot sensation. General soreness of posterior fauces and apparent extension of the irritation into the eustachian tubes. Smarting in the fauces. Sensation in the throat like that after eating chokepears. Difficulty of swallowing, which becomes so severe that he is unable to swallow even water.

*Every attempt to swallow is attended with excruciating pains through both ears.* Could not swallow because the throat felt so dry and rough.

### Causes and Aggravations.

In the beginning of the disease in cold weather, *cannot take hot fluids.* Cannot stand. When swallowing (pains up into ears and at root of tongue). *When rising up in bed* (faintness, dizziness.)

### Concomitants.

Much frontal headache, or pains in occiput. Shooting pains through *both ears,* worse when swallowing. Eustachian tubes feel obstructed.

Nose either obstructed and dry, or a watery discharge, or one nostril dry while mucus flows from the other. Acrid excoriating discharge (scarlatina), carotids or submaxillary glands swollen. Offensive smell from the mouth. Vomiting; albuminous urine. Stiffness of the neck. *Aching pain in lumbar region and extremities.*

*Feels sore all over from head to foot, especially muscular soreness. Groans when he moves, he is so sore* (*like Arnica*).

*Extreme prostration* from the beginning; cannot sit or stand, is so weak. *Restlessness and moaning from the general pains;* chilliness, especially at evening or night, followed by intense fever, the heat mostly in head, face, or upper part of body, while the extremities are cold.

It hastens suppuration (Janney) or aborts the whole trouble. Develops the eruption if there is one to be developed, other symptoms corresponding.

### Remarks.

This valuable remedy is one of the most frequently indicated in the beginning of diphtheria.

It has at this stage of the disease the severe pains and soreness of the head, back, and limbs more than any other remedy.

After the chilly stage the following heat is so much more prominent in the head, face, and upper part of the body, that when coupled with the sore, bruised feeling all over, it might be mistaken for an Arnica case if it were not for the *throat* symptoms which soon make their appearance; then the Arnica patient complains of the hard bed and wants to be moved to an easier place; while the Phytolacca patient is restless also, but moans every time he moves, he is so sore.

It is sometimes a little difficult to distinguish between this remedy and Bellad. on account of the intense fever of both, and the tendency of blood to the head; but the Bell. patient is more drowsy or inclined to stupor, with characteristic *starting from or when falling into sleep*, and the quick motions and delirium bordering on spasm, especially in children. The prostration also is not marked under Bell.

Apis has prostration at the beginning as marked as Phytol. Both have pains from throat up into ears, burning in throat, and albuminous urine; but the throat of Apis is more œdematous, and this œdema is all through the throat and into the *face*, especially around the eyes, and the burning in throat is coupled with the sharp, stinging pains so characteristic of Apis. Further comparison will show still further diagnostic differences between these three remedies, one of which is often indicated in the beginning of diphtheria.

The same symptoms must distinguish between these remedies in tonsillitis.

There is one affection in which I have found Phytol. exceedingly efficacious, of which I desire to make particular mention, viz.: chronic granular pharyngitis, and it is particularly in this trouble that the burning sensation, as of a hot substance in the throat, proves a genuine characteristic. In a public speaker the voice gives out, and the burning and soreness are greatly aggravated by making a plea (lawyer).

There are only two other remedies from which I have witnessed such markedly beneficial results in this kind of throat trouble. They are Arum. tri. and Merc. cyan. The indications for the former are too well known to need repeating here. In regard to the Merc. cyan. I have often found it promptly curative when the granulations seem inclined to break down into an ulcerated condition, or the posterior nares and back part of throat seem deprived in spots of mucous membrane. It is a peculiar appearance, more easily recognized than described. The sensation attending is *rawness or soreness*, like Causticum.

In regard to the doses of the remedies mentioned in this comparison in acute conditions, the Apis., Bell., and Phyt. will work well from the sixth to two hundredth or higher, according to susceptibility of patient.

In the chronic conditions I have used Phyt. in the CM. (Fincke) Merc. cyan.$^{30}$ or Arum. tri.$^{200}$ to $^{cm}$.

